# Integrative network analysis of miRNA-mRNA expression profiles during epileptogenesis in rats reveals therapeutic targets after emergence of first spontaneous seizure

**DOI:** 10.1038/s41598-024-66117-7

**Published:** 2024-07-03

**Authors:** Niraj Khemka, Gareth Morris, Laleh Kazemzadeh, Lara S. Costard, Valentin Neubert, Sebastian Bauer, Felix Rosenow, Morten T. Venø, Jørgen Kjems, David C. Henshall, Jochen H. M. Prehn, Niamh M. C. Connolly

**Affiliations:** 1 Centre for Systems Medicine & Dept. of Physiology & Medical Physics, RCSI University of Medicine and Health Sciences, Dublin, Ireland; 2 FutureNeuro SFI Research Centre, RCSI University of Medicine and Health Sciences, Dublin, Ireland; 3 Neuroscience, Physiology and Pharmacology, University College London, London, UK; 4 Epilepsy Center, Department of Neurology, Philipps University Marburg, Marburg, Germany; 5 Epilepsy Center Frankfurt Rhine-Main, Neurocenter, University Hospital Frankfurt and Center for Personalized Translational Epilepsy Research, Goethe-University, Frankfurt, Germany; 6 Interdisciplinary Nanoscience Center, Dept. of Molecular Biology and Genetics, Aarhus University, Aarhus, Denmark; 7 Omiics ApS, Aarhus, Denmark; 8 Division of Neuroscience, University of Manchester, Manchester, UK

**Keywords:** Epilepsy, Epileptogensis, microRNA, miRNA-mRNA interactions, Bayesian modelling, Temporal expression profiling, Epilepsy, miRNAs

## Abstract

Epileptogenesis is the process by which a normal brain becomes hyperexcitable and capable of generating spontaneous recurrent seizures. The extensive dysregulation of gene expression associated with epileptogenesis is shaped, in part, by microRNAs (miRNAs) – short, non-coding RNAs that negatively regulate protein levels. Functional miRNA-mediated regulation can, however, be difficult to elucidate due to the complexity of miRNA-mRNA interactions. Here, we integrated miRNA and mRNA expression profiles sampled over multiple time-points during and after epileptogenesis in rats, and applied bi-clustering and Bayesian modelling to construct temporal miRNA-mRNA-mRNA interaction networks. Network analysis and enrichment of network inference with sequence- and human disease-specific information identified key regulatory miRNAs with the strongest influence on the mRNA landscape, and miRNA-mRNA interactions closely associated with epileptogenesis and subsequent epilepsy. Our findings underscore the complexity of miRNA-mRNA regulation, can be used to prioritise miRNA targets in specific systems, and offer insights into key regulatory processes in epileptogenesis with therapeutic potential for further investigation.

## Introduction

Epilepsy is a chronic neurological condition, characterised by recurrent unprovoked seizures, which affects up to 50 million people worldwide^1^. Epileptogenesis is the process whereby a normal brain progressively becomes predisposed to spontaneous epileptic seizures, and can be triggered by a precipitating brain insult^2^. Typically, a delay or latent period of days to weeks exists between an initiating injury and the first spontaneous seizure. The mechanisms associated with epileptogenesis include select cell loss^3^, gliosis^4^, neuroinflammation^5^ and circuit restructuring^6^, as well as transcriptomic changes^7,8^. A better understanding of the mechanisms at play during epileptogenesis and consolidation of chronic epilepsy could provide biomarkers for those at risk of epilepsy development and progression to pharmacoresistance, and identify therapeutic targets that may delay, alleviate, or prevent epileptogenesis. A barrier would still remain, however, in the deployment of such treatments if identification of at-risk individuals must occur before the first clinical seizure. The identification of molecular targets that are still actionable after the earliest clinical signs (e.g., the first spontaneous seizure) would be an advantage in this respect.

MicroRNAs (miRNAs) are short non-coding RNAs (20–24 nucleotides in length) that, upon interaction with an argonaute protein, form an RNA-induced silencing complex which binds to complementary regions of target mRNAs, mainly in the 3’UTR^9^. MiRNAs thereby regulate gene expression by reducing transcript stability and/or repressing mRNA translation^10^. Due to imperfect base pairing between miRNAs and their mRNA targets, an individual miRNA can repress many different mRNAs, and individual mRNAs can be targeted by multiple miRNAs^10–13^. This multi-targeting property is appealing for anti-epileptogenesis therapy^14^ or for treating the complex and multifaceted pathophysiology of temporal lobe epilepsy (TLE), a highly pharmacoresistant epilepsy^15^. Indeed, many of the dysregulated gene networks in TLE are known or predicted targets of miRNAs and there is also extensive dysregulation of miRNA levels in experimental and human TLE^16–21^. This is further supported by experimental evidence that targeting miRNAs can have seizure-suppressive and anti-epileptogenic effects in rodents^8,22–26^. However, the complex regulatory networks of miRNAs greatly complicate interpretation of changes in the expression landscape downstream of miRNA dysregulation. Moreover, these interactions can be time-, cell type- and system-dependent, yet studies integrating miRNA or mRNA expression from the same individuals are still rare^27^. Identification of functional miRNA-mRNA interaction networks in specific biological systems is vital to better understand the miRNA regulatory environment^28^.

The functional annotation of miRNA-mRNA targeting often relies on computational prediction algorithms. Such methods are predominantly sequence-based, using the properties of mRNA targets and miRNA-mRNA binding sites (so-called ‘seed’ regions) to predict direct miRNA-mRNA binding interactions, but suffer from high false positive rates^29^ and do not consider miRNA or mRNA expression levels or the biological system under study. Furthermore, functional miRNA-mediated gene dysregulation is highly complex, with target site contexts that influence miRNA function including AU-nucleotide composition, RNA structure, cooperative action between proximal binding sites, supplementary miRNA:mRNA pairing beyond the seed region, and position of the target site within the UTR^9,13,30^. The effects of miRNA dysregulation may also extend beyond direct miRNA-mRNA binding interactions, for instance through miRNA-mediated suppression of transcription factors or other proteins with regulatory effects on gene expression^31,32^.

More recently, miRNA and mRNA expression profiles have been used to derive miRNA-mRNA networks via various computational approaches, ranging from expression correlation-based methods to advanced statistical methods including regression, mutual information, Bayesian network modelling and machine learning^33,34^. A Bayesian network is a graph-based probabilistic model that represents statistical dependencies between variables (e.g. miRNA and mRNA expression)^35^. Bayesian models can infer direct or indirect interactions and identify potentially causal relationships, which is not possible using correlation-based network inference algorithms alone. Bayesian networks have been successfully applied to infer interactions between molecular entities ranging from mRNA-mRNA interactions in the *S. cerevisiae* cell cycle^35^, to gene loci interactions contributing to human genetic diseases^36^. Bayesian networks have also been used to infer miRNA-mRNA interactions^37–39^.

Using the well-established rat perforant pathway stimulation (PPS) in vivo model of epileptogenesis^40^, we recently described time-course changes in functional (argonaute-loaded) miRNA expression^8^. This is a highly reproducible toxin-free model of epileptogenesis in which focal electrical stimulation of a specific neuroanatomical pathway is used to damage the hippocampus and generate neuropathology that closely matches human hippocampal sclerosis and is accompanied by spontaneous recurrent seizures. Here, we integrated this miRNA data with mRNA data from the same animals to construct temporal Bayesian networks of differentially expressed miRNA-mRNA-mRNA interactions through the progression of epileptogenesis and epilepsy emergence. To identify novel therapeutically viable miRNA and pathway targets, we analysed network properties to extract highly connected and central miRNAs and mRNAs which may strongly influence the epileptogenic process at each time-point. By integrating inferred network information with species conservation and human epilepsy-specific experimental data, we validated key miRNAs previously implicated in epilepsy and identified novel miRNAs of potential interest for therapeutic targeting.

## Methods and materials

### Data and code availability

The complete miRNA and mRNA sequencing datasets were previously published at the Gene Expression Omnibus (GEO) under accession no. GSE137473. The complete codes for model construction and network analyses are available at www.github.com/nirajkhe/EpimiRNA (10.5281/zenodo.7693342).

### Perforant pathway stimulation model of epilepsy

Tissue samples were obtained from the rat perforant pathway stimulation (PPS) model of epilepsy, as described previously^8,40^. This study analysed publicly available datasets. In the original study, experiments on rats were performed in accordance with the European Communities Council Directive (2010/63/EU) and were approved by the local regulation authority (Philipps University Marburg, Germany: Regierungspräsidium Gießen, 73/2013). Full experimental details are provided in Venø *et al*^8^. Briefly, male Sprague Dawley rats (weight 300–350 g) were equipped under anaesthesia with stimulation electrodes placed bilaterally in the angular bundle of the perforant pathway. One week after surgery, PPS was performed via continuous, bilateral 2 Hz paired-pulse stimuli, with a 40 ms interpulse interval, plus a 10 s train of 20 Hz single-pulse stimuli delivered once per minute. All pulses (0.1 ms duration) were delivered at 20 V. Stimulations lasted 30 min each on days 1 and 2, and 8 h on day 3. In this model, both hippocampal sclerosis (extensive neuron loss and attendant gliosis) and spontaneous recurrent seizures develop within a few weeks. Animals were euthanised by cardiac perfusion with ice cold 0.9% NaCl under deep anaesthesia with ketamine and xylazine at 24 h, 72 h and 10 days following PPS, within 24 h after the first spontaneous seizure (day of first seizure, DOFS), and 1 month following the emergence of spontaneous seizures (‘Chronic’), with 3 animals per time-point giving a total of 18 animals. Brains were rapidly removed and the entire hippocampus frozen and stored at -80 °C. Control rats received surgery but were not exposed to PPS and were euthanised the equivalent of 10 days after PPS. Ethics considerations prevented the use of multiple control timepoints.

### miRNA and mRNA sequencing

MiRNA sequencing used in this study was performed with the use of Ago2 immunoprecipitation (IP) to enrich for functional miRNAs, followed by RNA purification with Trizol (described in^8^). Ago2-IP RNA samples were prepared for sequencing using the TruSeq small RNA library prep kit (Illumina) and sequenced on an Illumina NextSeq 500 sequencer. Sequencing data was quality filtered using FastX-toolkit and adaptor trimmed using cutadapt^41^. Filtered data was mapped to mature miRNA sequences using Bowtie^42^. For mRNA sequencing (described in^8^), RNA samples were purified using Trizol and RNA quality was checked using the Agilent Bioanalyzer 2100. The RNA was depleted for ribosomal RNA using riboZero (Illumina) and sequencing libraries were prepared using ScriptSeq v2 (Illumina). Sequencing was done on an Illumina HiSeq4000 sequencer, and data was quality filtered and adaptor trimmed using trim_galore. Filtered data was mapped to the rat genome (rn6) using Tophat2. Transcripts were assembled and quantified using Cufflinks and Cuffnorm^43^. Visualization of data quality was done with fastqc.

### Differential expression and gene ontology enrichment analysis

miRNA and mRNA differential expression analyses were performed using DESeq2^44^ and Cufflinks (Cuffdiff)^43^ respectively, with an adjusted p-value cut-off (Benjamini & Hochberg, BH) of 0.05. Differentially expressed miRNA and mRNA were identified at each time-point with respect to control (10 days after treatment). Gene ontology enrichment analysis of differentially expressed mRNA was performed using the ClusterProfiler R package^45^ with an adjusted p-value cut-off (BH) of 0.05 with at least 5 genes per term. All dysregulated miRNAs and mRNAs are listed in Supp. Tables 1a,b.

### SAMBA bi-clustering

A total of 3986 unique mRNAs were differentially expressed over the time-course. To reduce the number of parameters (mRNAs) for Bayesian modelling, we used SAMBA (Statistical and Algorithmic Method for Bi-cluster Analysis) bi-clustering to filter the most relevant mRNAs for each time-point^46^. SAMBA models the input dataset as a bipartite graph, where one node type corresponds to genes/mRNAs and the second node type corresponds to experimental conditions (time-points), and finds complete bipartite subgraphs composed of gene nodes with bounded degree ^46^. SAMBA identifies modules of mRNAs that show significantly correlated expression patterns across time-points/conditions. Unlike conventional clustering (e.g. hierarchical clustering), bi-clustering allows mRNAs to be clustered in more than one module. SAMBA bi-clustering was performed using Expander with 10% overlap between identified modules at each time-point^47^. To obtain a single set of mRNAs for each time-point, mRNAs/clusters identified in that time-point were merged. Bi-clustered mRNAs are indicated in Supp. Table 1a.

### Bayesian network development

**Figure 1:**
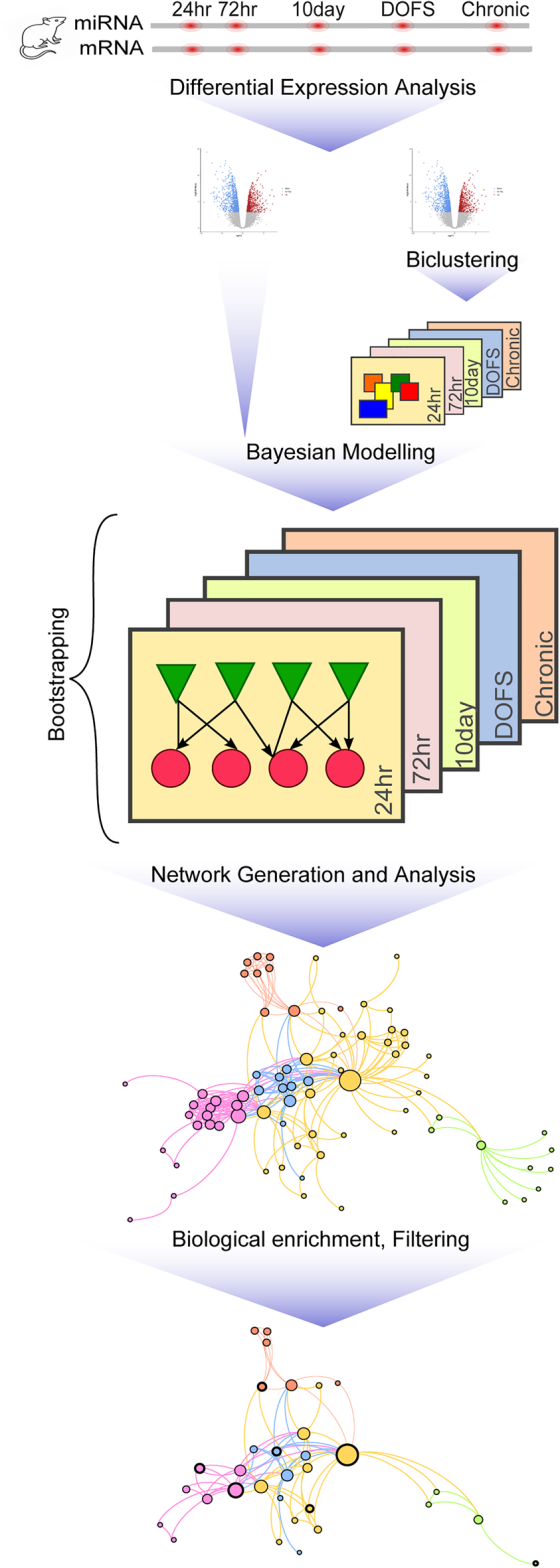
Workflow of Bayesian model development and network analysis. miRNA and mRNA expression profiles from hippocampal tissue of rats exposed to perforant path stimulation (PPS) were obtained at 5 time-points (n = 3 animals from 24 h, 72 h and 10 days following PPS, within 24 h after the first spontaneous seizure [day of first seizure—DOFS], and 1 month following emergence of spontaneous seizures [Chronic]). Differentially expressed miRNAs and mRNAs (following SAMBA bi-clustering to subset the most relevant mRNAs) were integrated to construct a Bayesian network for each time-point, and analysis was performed across all networks. Finally, network inference was enriched with biological data to filter the networks and identify critical miRNAs and their mRNA targets.

The workflow schema of the model is shown in Fig. 1. Model development was based on the approach of ^38^. The expression values of each differentially expressed miRNA and bi-clustered mRNA were z-score normalised and used as model input (three replicates over five time-points, for a total of 15 measurements). The number of differentially expressed miRNAs and mRNAs at each time-point is given in Table 1. The miRNA and bi-clustered mRNA expression profiles were integrated in a Bayesian model for each time-point. As the prior knowledge of miRNA-mRNA interaction networks in epileptogenesis is far from complete, a prior network structure was not introduced, and the network structure was learned from the input expression profiles of miRNA and mRNA at each time-point. Bayesian models were constructed for each time-point using the Hill Climbing algorithm from the ‘bnlearn’ R package and the Bayesian information criterion (BIC) score^48^. Given the small sample size, bootstrapping (resampling with 1000 replicates) was applied to infer a more robust structure. The inferred probabilistic networks contain directed edges (interactions) between miRNA-miRNA, miRNA-mRNA, mRNA-miRNA, and mRNA-mRNA. A strength score was assigned to each edge based on the empirical frequency of each edge from the set of networks learned from bootstrapping. To identify network features that are most pronounced at each time-point, only edges in the highest quartile of strength scores for all edges at that time-point were retained.

**Table 1:** Number of differentially expressed miRNAs and mRNAs at each time-point.

	24 h	72 h	10 day	DOFS	Chronic	Total
miRNA (DESeq2)	17	26	27	158	82	189
mRNA (Cufflinks)	2153	925	1020	2778	138	3986
mRNA (SAMBA)	109	191	221	136	23	489

The method used to determine differential expression is listed in brackets. The numbers of differentially expressed mRNAs before (Cufflinks) and after SAMBA bi-clustering are shown. The Total column indicates the number of unique differentially expressed miRNAs/mRNAs across the timecourse.

*DOFS* day of first seizure.

### Network analysis

Gephi (v0.9.7) was used to visualise and analyse the networks^49^. Network statistics (degree, betweenness centrality) were calculated using the igraph package (v1.2.3, https://igraph.org/r/) in R. Figures were generated in R using ggplot2, ComplexHeatmap and tidyr/plyr packages^50,51^. Upset plots were generated in MATLAB R2017A and Fig. 4E was visualised using Cytoscape V3.8.0^52^. Epilepsy-related genes were collated from Wang *et al*^53^ and the DisGeNET^54^, epiGAD^55^, CARPEDB (http://carpedb.ua.edu/) and comparative toxicogenomics databases (CTD; curated genes only)^56^.

### Comparison of inferred networks with publicly available resources

MiRNA sequences were extracted from miRBase^57^ and predicted human miRNA-mRNA target interactions (MTIs) with confidence class (Very High, High, Medium, Low) were extracted from miRDIP^58^. Experimentally validated MTIs (human, mouse, rat) were extracted from miRTarBase V9^59^ and TarBase V8^60^. mRNA and small non-coding RNA differential expression data from human hippocampal tissue resected from mesial temporal lobe epilepsy patients^27^ were kindly provided by Dr. James Mills (miRNA p < 0.06, mRNA p < 0.05). The human iCLIP dataset of Ago2-bound RNAs from resected hippocampal slices from treatment-resistant epilepsy patients was available in-house^61^. For comparison with publicly available resources, inferred MTIs containing miRNAs with no corresponding human (hsa) miRNA or genes not mapped to official gene symbols (gene names containing “.”, LOC*, or RGD*) were removed. Three rat gene symbols were changed to human synonyms/orthologues (*Cecr6*/*TMEM121B*, *Pnmal2*/*PNMA8B* and *Oasl2*/*OASL*).

## Results

### mRNA bi-clustering identifies stage-specific processes underlying epilepsy development

**Figure 2:**
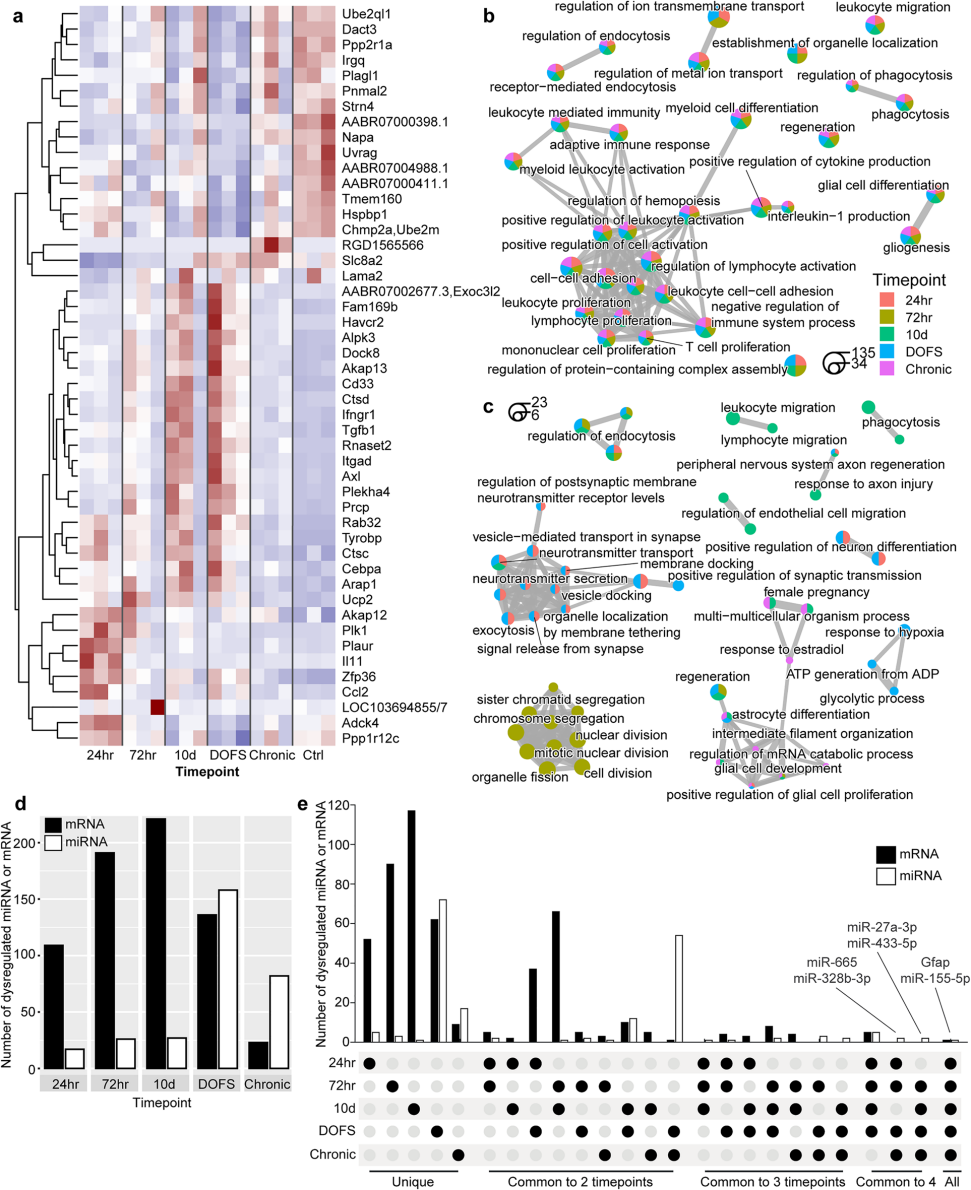
Temporal mRNA and miRNA dysregulation in the rat PPS model of epilepsy. (**a**) Expression of the 15 most significantly dysregulated mRNAs from each time-point. DOFS; day of first seizure. All dysregulated mRNAs are listed in Supp. Table 1a and visualized in Supp. Fig. 1. (b.c), Gene ontology enrichment analysis (biological processes, BPs) of (**b)** all differentially expressed, and (**c**) SAMBA-bi-clustered mRNAs at each time-point. The 10 most significantly enriched BPs (adjusted p < 0.05) from each time-point are shown. Similar BPs are grouped together, and some terms have been removed for clarity. Nodes are coloured according to the time-points at which they were significantly enriched, and node size indicates the number of dysregulated genes associated with each BP. (**d**) Number of differentially expressed (DE) miRNAs and bi-clustered mRNAs at each time-point. (**e**) Upset plot showing common and unique DE miRNAs and bi-clustered mRNAs at each time-point.

We recently sequenced Ago2-loaded miRNA from rat hippocampi at multiple time-points during epileptogenesis, using the perforant path stimulation (PPS) model^8^. This identified 189 dysregulated miRNAs (up- or down-regulated relative to control) across the time-course of epilepsy development and into the chronic epilepsy phase. Notably, the PPS model showed much lower variability of miRNA dysregulation between biological replicates than other models used in the same study, thereby facilitating detailed molecular network modelling with the given dataset^8^. To explore the effects of the multitude of miRNAs dysregulated in this model, we here analysed mRNA from the same animals at each time-point. The available data included a total of 1.4 billion reads which were sequenced with an average of 78 million reads per sample. These reads were processed to identify differentially expressed (DE) mRNAs at each time-point. Gene dysregulation (Fig. 2a, Supp. Fig. 1, Supp. Table 1a) indicated different pathological molecular mechanisms underlying early (24 h after PPS) and late (72 h, 10 day) epileptogenesis, as well as following epilepsy emergence [day of first seizure (DOFS), 1 month after seizure (Chronic)]. Gene ontology enrichment analysis (biological processes) of DE mRNAs at each time-point identified wide-ranging and expected terms associated with epileptogenesis including terms related to immune response, differentiation/development, proliferation/migration, apoptosis, metabolism, and transport (Fig. 2b, Supp. Table 2a). Immune dysregulation particularly is well established in both experimental and human epilepsies^5,27,62^. However, the top enriched terms were identified at most/all time-points (Fig. 2b), and such commonality can confound identification of critical or novel stage-specific mechanisms underlying pathogenesis. To reduce the number of functionally correlated mRNAs, we performed SAMBA bi-clustering^46^ which groups modules of mRNAs with correlated expression patterns across conditions, identifying mRNAs related to specific time-points (Methods). Bi-clustering reduced the number of unique DE mRNAs across all time-points from 3986 to 489 (Table 1, Supp. Table 1a, Supp. Fig. 1). Gene ontology enrichment of the bi-clustered mRNAs identified time-point specific processes (Fig. 2c, Supp. Table 2b) including cell cycle and migration processes during late epileptogenesis (72 h, 10 day), and the emergence of energy and metabolic processes at spontaneous seizure onset (DOFS). Interestingly, multiple enriched processes overlapped between the 24 h and DOFS time-points including synapse, synaptic vesicle, and neurotransmitter-related terms, indicating mechanisms likely directly associated with circuit excitability and seizure occurrence.

We next looked at the number of miRNAs and bi-clustered mRNAs dysregulated at individual or multiple time-points. This revealed major differences over the course of epilepsy. Most notably, the number of dysregulated miRNAs remained relatively low during phases of epileptogenesis, then increased substantially at the time when animals experienced a first spontaneous seizure (DOFS; Fig. 2d, Supp. Table 1b). The effect of this was a closer ratio of dysregulated miRNAs/mRNAs upon establishment of a functioning, hyperexcitable network that generates spontaneous seizures. At the chronic time-point (one month after epilepsy was established) the number of dysregulated miRNAs had decreased, although the proportion of dysregulated miRNAs/mRNAs had not returned to that observed in the earlier phases of epileptogenesis. These two epilepsy time-points (DOFS, Chronic) also shared the highest number of dysregulated miRNAs (54, Fig. 2e), in line with the known key role of the miRNA system in epilepsy, and potentially indicating long-term establishment of a new brain state. In contrast, the 72 h and 10 d pre-epileptic time-points shared the highest number of dysregulated mRNAs (66). The 24 h and DOFS time-points shared 37 mRNAs, again suggesting seizure-related processes. A single miRNA (rno-miR-155-5p) and single mRNA (*Gfap*) were dysregulated at all five time-points. Thus, the molecular landscape is most disrupted upon the emergence of spontaneous seizures and changes evident throughout the time-course indicate common and distinct processes underlying this progression.

### Temporal miRNA-mRNA-mRNA Bayesian networks of epilepsy development

**Figure 3:**
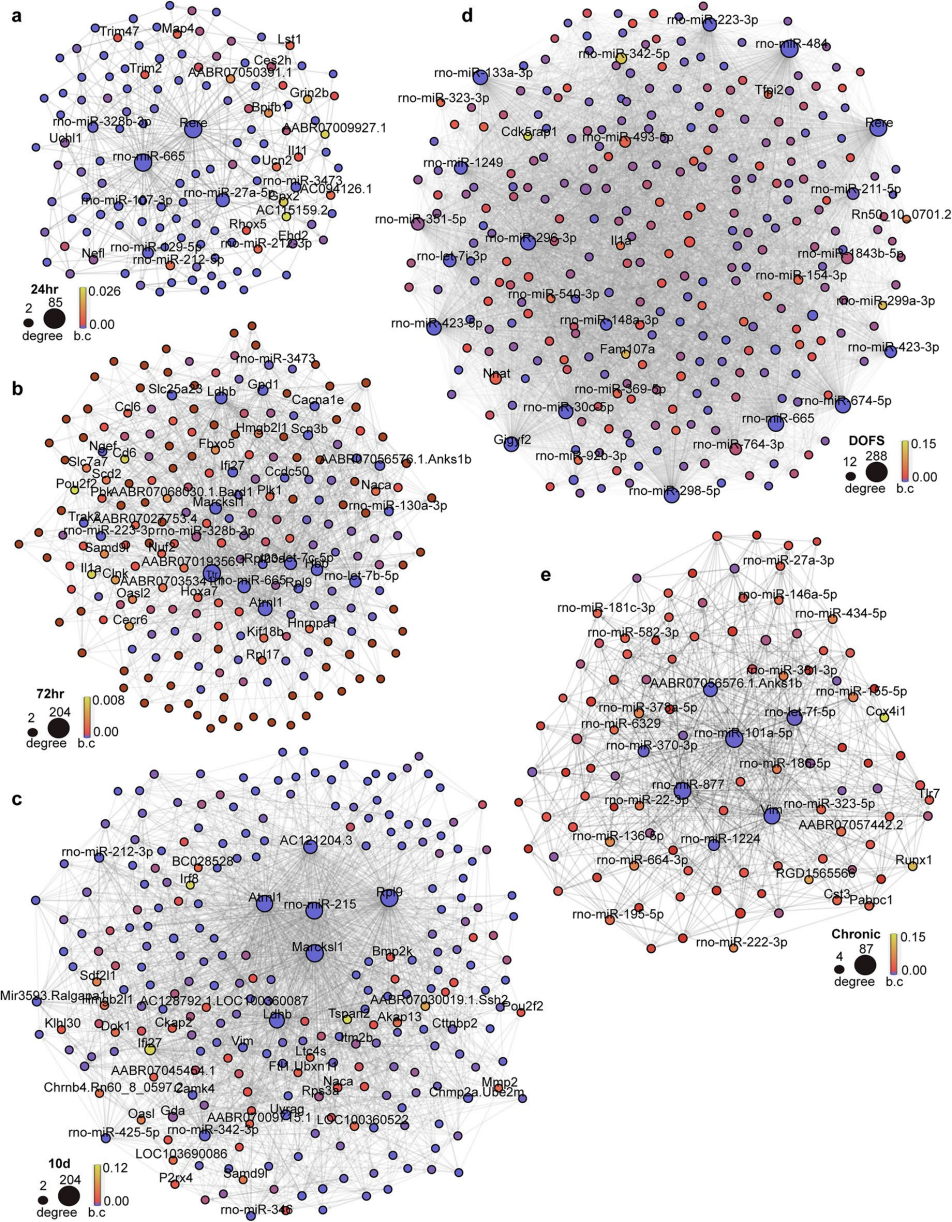
miRNA-mRNA-mRNA Bayesian directed networks for each time-point: (**a**) 24 h, (**b**) 72 h, (**c**) 10 day, (**d**) day of first seizure, DOFS, (**e**) Chronic. Dysregulated miRNAs or mRNAs are represented by circles (nodes), and nodes with an inferred interaction are connected by edges. Node size indicates the degree of connectivity of the node within each network, and node colour indicates betweenness centrality (b﻿.c). The top 20% of nodes for each network statistic are labelled. All network interactions are listed in Supp. Table 3.

A Bayesian network is a probabilistic formalism applied to infer gene regulatory networks based on conditional dependence between variables (here miRNAs and mRNAs)^63^. To identify functional molecular interactions underlying the rodent model of epileptogenesis and seizure emergence, we utilised the differentially expressed miRNA and bi-clustered mRNA profiles to generate Bayesian miRNA-mRNA-mRNA networks at each time-point (Fig. 3a–e, Supp. Table 3). The directed networks captured direct and indirect interactions between miRNAs and their mRNA targets, and also included miRNA-miRNA, mRNA-mRNA, and mRNA-miRNA interactions.

**Figure 4:**
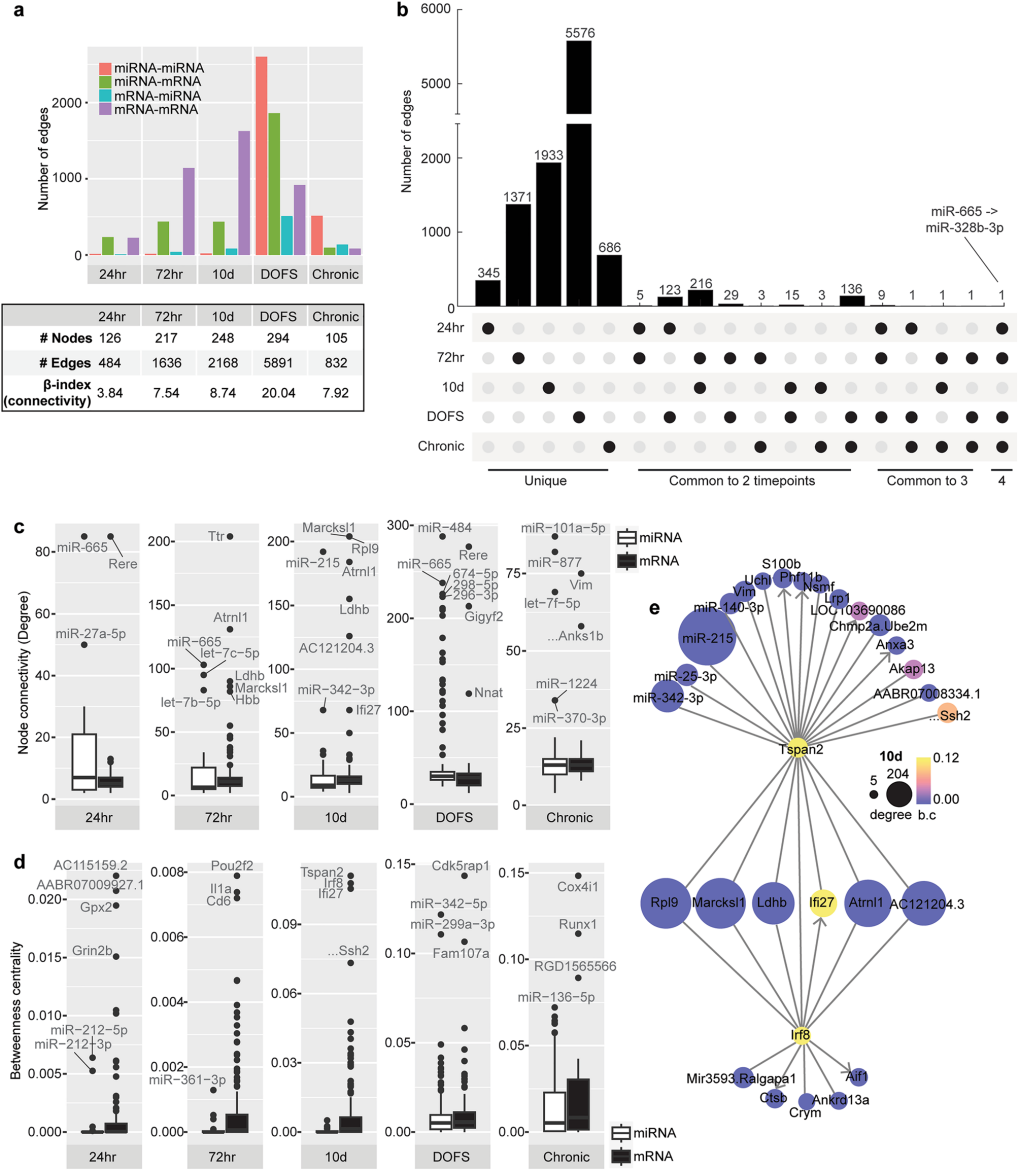
Network properties for each time-point. (**a**) Number of edges (interactions) per time-point, split by interaction type. The table shows the total number of nodes and edges. β-index (connectivity measure) = #edges/#nodes. (**b**) Upset plot showing the number of common and unique interactions at each time-point. The y-axis is cut for clarity. (**c**) Connectivity degree (incoming + outgoing edges) of miRNAs and mRNAs per time-point (d) Betweenness centrality of miRNAs and mRNAs per time-point. (**e**) Tspan2 and Irf8 connections at the 10d time-point. Target nodes are indicated with arrowheads (arrowheads were removed from Irf8 and Tspan2 for clarity). b.c betweenness centrality.

The number and type of interactions at each time-point (Fig. 4a) demonstrated that the early and later epileptogenesis time-points (24 h, 72 h, 10 d) were predominated by canonical interactions (miRNA-mRNA, mRNA-mRNA), but that emergence of the first spontaneous seizure (DOFS) was associated with excessive network disruption, some of which remained up to one month after the emergence of seizures (Chronic). Indeed, the DOFS time-point was characterised by excessive connectivity (Fig. 4a). Looking at shared network interactions across time-points (Fig. 4b), the 72 h and 10 d latent phase time-points had the highest number of interactions in common (216). The epilepsy time-points (DOFS, Chronic) also shared a high number of interactions (136), and seizure-related processes were again indicated by shared interactions between the 24 h and DOFS time-points (123).

We next investigated node connectivity and centrality for each network (Supp. Table 4). The degree of a node is the number of in-coming and out-going edges of that node, with highly connected nodes, or hubs, considered essential for network connectivity. Nodes in the DOFS network had higher overall degree indicating increased connectivity (Fig. 4c), with most high degree nodes being miRNAs, suggesting their influence at this juncture. Rno-miR-665, dysregulated at multiple time-points (24 h, 72 h, DOFS, Chronic) and involved in neuroprotection^64^, was highly connected throughout the time course. Other miRNAs were highly connected at individual time-points only (*e.g.,* rno-miR-27a-5p at 24 h; rno-miR-484 and rno-miR-674-5p at DOFS; rno-miR-101a-5p at Chronic). Interestingly, several of the most highly connected mRNAs have been associated with epilepsy and other neurodegenerative disorders [*e.g., Rere, Marcksl1, Ttr*^65–67^ and Discussion], supporting the biological relevance of our approach.

Betweenness centrality is a measure of how often a node appears in the shortest path between two other nodes^68^. A node with higher betweenness centrality can be expected to have a significant role in the propagation of effects across the network. Interestingly, the network’s most highly connected nodes were not the most central (Fig. 4d), indicating that while these nodes may not have direct interactions with many other nodes, they may be important in connecting the whole network. At the 10 d time-point, for instance, *Tspan2* (tetraspanin 2) and *Irf8* (interferon regulatory factor 8) were the two most central nodes despite having relatively few connections (23 and 12 respectively, Supp. Table 4). Rather, these nodes provide interconnections between several of the most highly connected nodes at this time-point (Fig. 4e).

### Enrichment of inferred network interactions with biological data predicts critical epileptogenesis-associated miRNA-mRNA interactions

**Figure 5:**
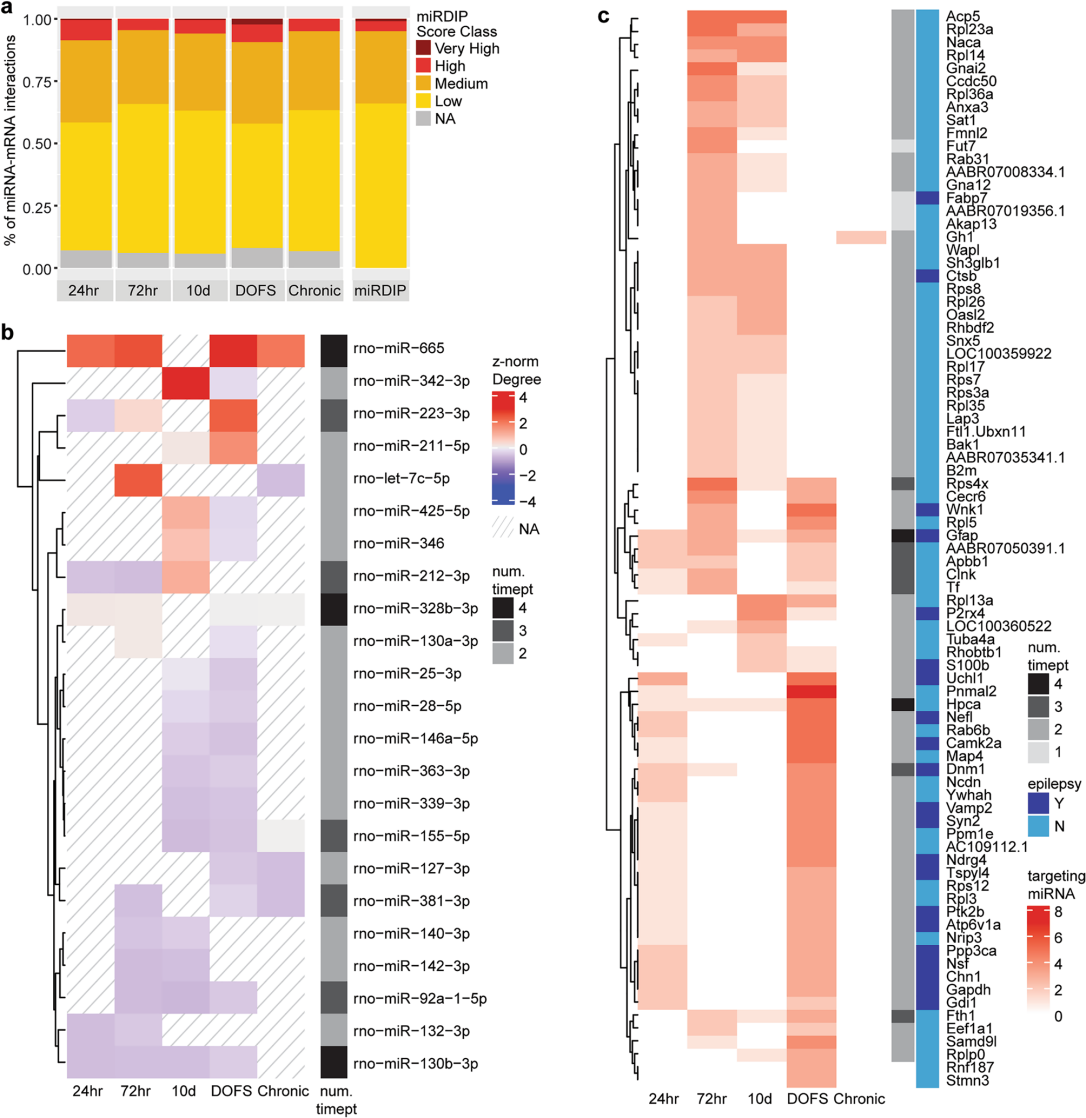
Analysis of the network-inferred miRNA-mRNA interactions. (**a**) Proportion of inferred miRNA-mRNA interactions that are computationally predicted (miRDIP) at various confidence classes. NA indicates network interactions not present in the miRDIP database. (**b**) The degree (number of mRNA targets) of 23 short-listed miRNAs with inferred miRNA-mRNA interactions at > 1 time-point (num. timept). The degree is z-score normalised along time-points – the miRNA with most mRNA targets at each time-point is dark red. Hatched fill indicates that the miRNA had no inferred mRNA targets at that time-point (NA). miRNAs were clustered by hierarchical clustering. (**c**) mRNAs dysregulated in > 1 time-point and targeted by > 2 of the 23 short-listed miRNAs across the time-course. Red shading indicates the number of miRNAs targeting each mRNA at each time-point.

We next used these networks to predict miRNAs and canonical miRNA-mRNA interactions with high impact on the progression of epileptogenesis and spontaneous seizure emergence. We therefore extracted the 189 dysregulated miRNAs from all time-points along with their differential expression across the time-course, network characteristics (degree and betweenness centrality), sequence conservation, and network-predicted mRNA targets (Supp. Table 5). We excluded 55 miRNAs with no inferred mRNA targets across the time-course, and a further 13 with no corresponding human orthologue. We then compared the network-inferred miRNA-mRNA interactions for the remaining 120 miRNAs with computationally predicted miRNA-mRNA interactions from miRDIP^58^; Fig. 5a]. There was a higher proportion of Medium and High confidence interactions in the Bayesian network compared to all miRDIP interactions for these miRNAs. The DOFS time-point also had a higher number of Very High confidence interactions. The Bayesian network also predicted miRNA-mediated indirect interactions (*e.g.*, transcription factor regulation of target mRNAs), providing information additional to that from sequence-based predictions alone.

We further filtered the miRNAs to retain the 26 miRNAs with inferred mRNA targets at multiple time-points (to increase robustness of findings and to focus on those miRNAs that may have significant impact across the disease course). Of these, 23 miRNAs (Supp. Table 5) had a seed sequence (nt 2–8) conserved from rat to human (excluding rno-miR-496-3p, rno-miR-21-3p, and rno-miR-361-3p), increasing potential translational relevance. Three miRNAs (rno-miR-130b-3p, rno-miR-328b-3p, rno-miR-665) had mRNA targets at four time-points, with rno-miR-665 having the highest number of targets at all four time-points (Fig. 5b). The majority of these miRNAs were up-regulated in the PPS model, although rno-let-7c-5p and rno-miR-346 were down-regulated (Supp. Table 5^8^). Some miRNAs derive from the same miR families, including the miR-25 family (rno-miR-25-3p, rno-miR-92a-1-5p), the miR-130 family (rno-miR-130a-3p, rno-miR-130b-3p), and the miR-132 family (rno-miR-132-3p, rno-miR-212-3p) (Supp. Table 5). Several of these short-listed miRNAs have been previously associated with epilepsy(^19,69,70^ and Discussion). Nevertheless, we identified several additional miRNAs which have not yet been studied in epilepsy, offering a list of novel potential therapeutic miRNA targets.

**Figure 6:**
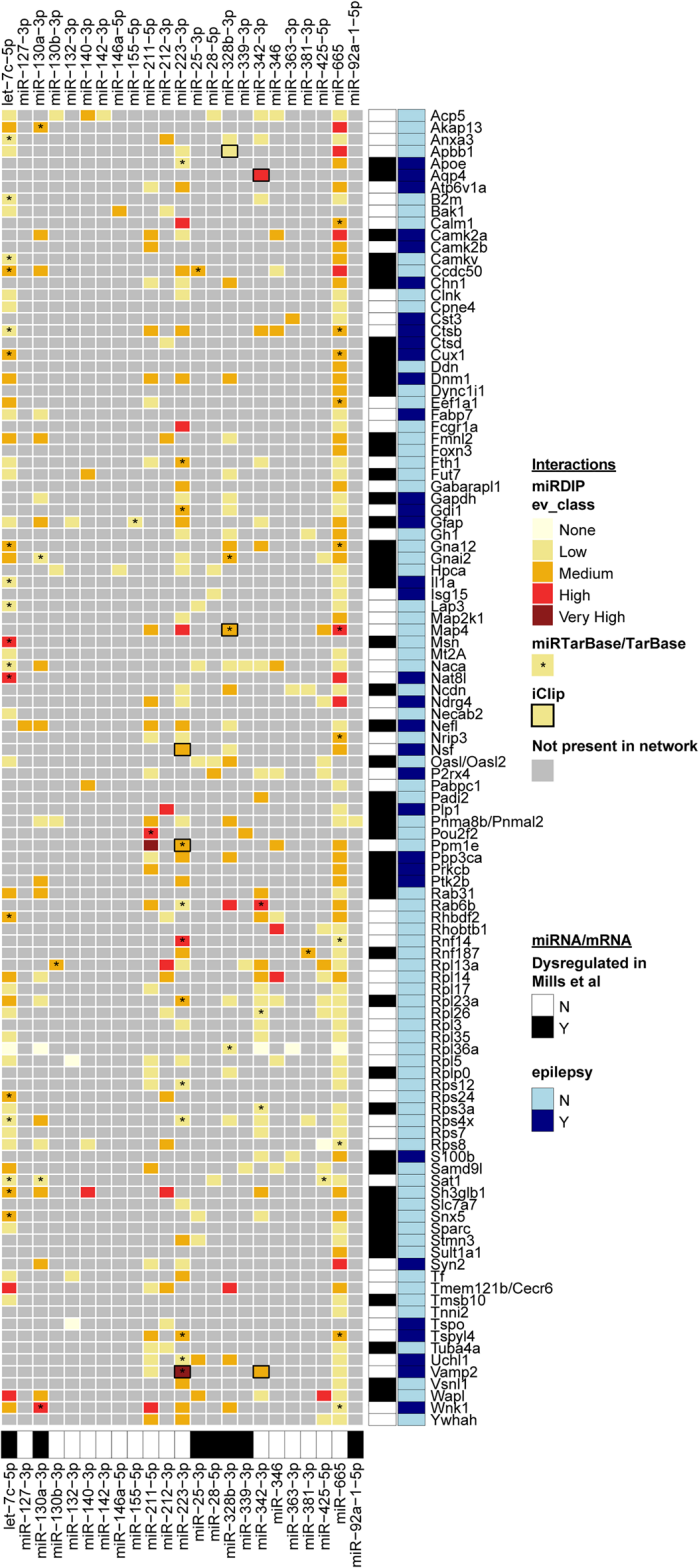
Comparison of the filtered miRNA-mRNA interaction network with human data. Visualisation of the 23 miRNAs, 110 mRNAs, and 398 unique miRNA-mRNA target interactions (MTIs; non-protein coding mRNAs removed) in the filtered inferred network across all time-points, and alignment with several publicly available resources. MiRNAs or mRNAs dysregulated in human temporal lobe epilepsy^27^ are indicated by black shading below and to the right of the heatmap, respectively. mRNAs previously implicated in epilepsy are indicated by navy annotations to the right of the heatmap. Inferred MTIs are coloured according to miRDIP confidence level. MTIs shaded grey do not occur in the inferred network. MTIs with experimental evidence (miRTarBase^59^, TarBase^60^) are indicated by *. MTIs present in the human iCLIP dataset^61^ are indicated by a solid black border.

Finally, to explore the targeting of these 23 miRNAs across the time-course, we extracted their inferred mRNA targets dysregulated at > 1 time-point. These 436 miRNA-mRNA target interactions (MTIs) and the mRNA targets are listed in Supp. Table 6ab and visualised in Fig. 6. The mRNAs targeted by > 2 of the 23 miRNAs are shown in Fig. 5c. The Chronic time-point has just a single mRNA, *Gh1*, in this heatmap, indicating that the 23 shortlisted miRNAs may be more active/have more impact at the epileptogenic time-points prior to epilepsy establishment. A group of mRNAs are targeted both at 24 h and DOFS, again representative of seizure-related processes, and many of these have previously been implicated in epilepsy (Fig. 5c). However, the group of mRNAs targeted at both 72 h and 10 d suggests a set of miRNA-mRNA interactions that may be specifically involved in the transition from precipitating injury to the emergence of chronic epilepsy, including several ribosomal proteins (*Rpl, Rps)*.

To increase the translational relevance of this filtered network, we compared the miRNAs, mRNAs and miRNA-mRNA interactions with several publicly available resources, including two relevant human hippocampal datasets from epilepsy patient samples (Fig. 6, Supp. Table 6a). We compared the miRNAs and mRNAs with dysregulated miRNAs and mRNAs identified in human hippocampal tissue resected from mesial temporal lobe epilepsy patients^27^. Seven of the 23 miRNAs and 48 of the 110 mRNAs were in common (Fig. 6, black annotations), indicating close agreement with human data. We compared the inferred MTIs with several publicly available resources – computationally-predicted human MTIs from miRDIP^58^, experimentally validated MTIs from miRTarBase and TarBase^59,60^, and a human iCLIP dataset of Ago2-bound RNAs from resected hippocampal slices from treatment-resistant epilepsy patients^61^. The alignment of our network with these resources is visualised in Fig. 6, and enables prioritisation of MTIs of interest. For instance, the miRNA rno-let-7c-5p is dysregulated in human epilepsy and inferred mRNA targets present in multiple other resources include *Cux1* (Cut like homeobox 1), *Il1a* (Interleukin 1 Alpha), *Msn* (Moesin), *Nat8l* (N-acetyltransferase 8 like) and *Wapl* (WAPL cohesin release factor). The targeting of *Cux1*, *Il1a, Msn* and *Nat8l* by let-7c-5p have already been experimentally validated. Furthermore, the targeting of *Vamp2* (Vesicle associated membrane protein 2), *Aqp4* (Aquaporin 4) and *Map4* (Microtubule associated protein 4) by miR-223-3p, miR-342-3p and miR-328b-3p respectively were also observed in the iCLIP dataset, validating these network-inferred MTIs as functional interactions in human epileptic tissue. The MTI miR-223-3p-*Vamp2* is predicted with very high confidence and has been experimentally validated, and could therefore be of particular interest. Taken together, this alignment enriches our inferred network with publicly available and human data, and further informs the identification of both novel and previously-implicated miRNAs, mRNAs and MTIs for further research in epileptogenesis.

## Discussion

While extensive dysregulation of miRNAs and mRNAs in human temporal lobe epilepsy and associated animal models has been well documented^8,19,71^, the interaction of these two RNA landscapes and the temporal progression of their interactions during epileptogenesis is yet to be thoroughly explored. In this study we utilised temporal miRNA and mRNA expression profiles from rat hippocampi following perforant path stimulation (an established model of epileptogenesis)^40^ to construct directed Bayesian networks of miRNA-mRNA-mRNA interactions at multiple time-points throughout epileptogenesis and the emergence of spontaneous seizures into the chronic epilepsy phase.

We first analysed the mRNA expression time course and identified significant and disparate dysregulation at all time-points (we previously reported extensive analysis of the miRNA expression profile^8^). Broad dysregulation of mRNA expression during epileptogenesis and epilepsy^18,71–73^ is indicative of the network disruption and circuit restructuring evident during epileptogenesis^8^, and can confound identification of the critical molecular pathways regulating these processes. We performed SAMBA bi-clustering^46^ to filter the most relevant mRNAs by identifying subsets of mRNAs highly correlated with each time-point. Bi-clustering resulted in clear separation of time-point-specific gene ontology terms (Fig. 2c), including cell-cycle and migration processes during early (72 h) and late epileptogenesis (10 d) respectively, which may be reflective of anatomical and functional changes associated with the development of epilepsy. Perhaps surprisingly, only a single miRNA (rno-miR-155-5p) and single bi-clustered mRNA (*Gfap*) were dysregulated at all time-points, further indicating disparate processes underlying various stages of epileptogenesis. *Gfap* is a predominantly astrocytic gene with strong links to epilepsy^74^, and astrocytosis, which is associated with increased expression of *Gfap*, is the most consistent finding in resected hippocampi from patients with treatment-resistant epilepsy and ´hippocampal innate inflammatory gliosis only´ on histopathology^75^. Moreover, astrogliosis alone is sufficient to generate epilepsy in rodents^76,77^. miR-155-5p is up-regulated in human and animal TLE^78^ and its inhibition mediates neuroprotective and anti-seizure effects which are associated with reduced inflammation and oxidative stress^78,79^.

The complexity of miRNA-mRNA targeting and associated gene-regulatory networks has been well documented^10,13^, yet the elucidation of system-specific functional miRNA-mRNA interactions is vital to enable a detailed understanding of pathophysiological molecular mechanisms and putative therapeutic targets. Here, we applied an established approach to integrate miRNA and mRNA data to infer miRNA-mRNA regulatory modules^38^ and applied this for the first time in the context of epileptogenesis. Our expression-based network captures both direct and indirect bidirectional interactions between miRNA and mRNA, as well as miRNA-miRNA and mRNA-mRNA interactions. While the Bayesian approach implemented here can provide evidence of causality, very large sample sizes and incorporation of prior knowledge are necessary to identify truly causal associations in complex networks^80^. Other studies have combined expression-based inference algorithms with sequence-based predictions^34,81^. We did not incorporate sequence-based information at the network-building stage, to enable identification of both direct and indirect inferred interactions. Following network generation, we compared inferred MTIs with computationally predicted MTIs from the miRDIP database. MTIs with High/Very High confidence in miRDIP could be interesting interactions to explore.

Among different time-points analysed in this study, the 24 h and DOFS time-points were similar in several aspects, including bi-clustered dysregulated mRNA, pathway enrichment terms, inferred network interactions, and inferred mRNA targets of our shortlisted miRNAs. It is likely that these commonalities are associated with seizure occurrence. At the 24 h time-point, these probably reflect evoked activity, an acute response to the prolonged stimulation of the afferent pathway to the hippocampus that is the epileptogenic insult in this model. High levels of dysregulation were particularly evident at DOFS, indicating that the emergence of spontaneous seizures is associated with excessive network disruption, more so than the occurrence of a standalone (electrically induced) seizure. This enrichment of changes during the DOFS is also evident for other forms of noncoding RNA in the same model^82^. This has implications for the development of disease-modifying treatments. Currently, deployment of an anti-epileptogenic therapy requires the identification of at-risk individuals using biomarkers of a clinically-silent state (the latent phase of epileptogenesis)^83^. No such biomarkers have been validated, and the timing of any intervention in the hours, days or weeks after a precipitating injury is unknown. Without biomarkers, there would be a risk of treatments being given to individuals who would not have developed epilepsy^84^. If substantial changes to the gene expression landscape are ongoing during and immediately after a first spontaneous seizure, then disease-modifying treatments to prevent chronic epilepsy may be successful when given after the first clinical signs of epilepsy, avoiding the restrictions implied by the need to identify and act on earlier phases of the epileptogenesis process. Thus, future anti-epileptogenic interventions could be disease-modifying if deployed against processes active at and directly after the DOFS time-point.

Following network construction, we performed network analysis to identify high-impact miRNAs and their mRNA targets with potential therapeutic relevance in epilepsy. Several highly connected mRNAs have been associated with epilepsy and other neurodegenerative disorders. *Rere* (Arginine-glutamic acid dipeptide repeats protein) over-expression triggers apoptosis^85^ and *Rere* mutations are associated with neurodevelopment disorders and seizures^65^. *Marcksl1* (MARCKS like 1, synonym MLP) is dysregulated in the hippocampus following kainic acid-induced seizures^67^, while Rpl9 (Ribosomal protein L9) was up-regulated in microglia samples from kainic acid-treated mice^86^. Ttr (Transthyretin) is involved in ABeta aggregation and is dysregulated in Alzheimer’s and Parkinson’s disease^66^. Interestingly, mRNA nodes were generally more central than miRNA nodes, potentially indicating that mRNA may be more responsible for the propagation of effects across the network.

An ongoing challenge in the area of multi- ‘omics and gene–gene network inference is the identification of underlying molecular mechanisms and putative therapeutic targets from large networks. Here, to screen miRNAs of highest potential impact and translational relevance, we combined differential expression analysis, network inference, and sequence conservation information to derive a short-list of miRNAs that a) were dysregulated at multiple time-points (to increase robustness and exclude potentially aberrantly dysregulated miRNAs), b) had inferred miRNA-mRNA interactions at multiple time-points, c) had human miRNA orthologues with conserved seed region sequences. The 23 short-listed miRNAs (Fig. 5b, Supp. Table 5), include several miRNAs previously associated with epilepsy, lending physiological support to our approach. miR-132-3p has been strongly implicated in epilepsy, due to its interaction with the transcription factor CREB^19,69,70,87,88^. It was recently found to be overexpressed in glia in human and rat TLE and may be a therapeutic target in astrocytes^89^. miR-146a-5p is another predominantly astrocytic miRNA, and has been associated with epilepsy as it regulates neuroinflammation^19,69,70,87,88,90^. miR-212-3p is dysregulated in multiple pre-clinical epilepsy models^8,19,69^, while miR-25-3p was found to reduce epileptiform discharges in primary cultured neurons^91^. Other epilepsy-implicated miRNAs in our short-list include miR-130a-3p^19,70^, miR-155-5p^87^, miR-223-3p^92^ and miR-328(b)-3p^20^. The short-listed miRNAs also include miRNAs with a putative novel role in the disease. These novel miRNAs have inferred mRNA targets that have been previously associated with epilepsy (Fig. 5c, Supp. Table 6b), offering insights into their potential roles.

Our screening process by necessity excludes some miRNAs. For example, our method excludes potentially high-impact miRNAs with high centrality and/or high degree at single time-points only, such as rno-miR-136-5p, rno-miR-212-5p (most central nodes at DOFS and Chronic time-points respectively) and rno-miR-278a-5p (central and highly connected at Chronic time-point). Nevertheless, it may be more difficult to time the targeting of nodes dysregulated at single time-points. Furthermore, perturbation of highly connected and highly central nodes (including rno-miR-328b-3p in our short-list) may broadly disrupt the network and the implications of this should be carefully considered. Interestingly, hsa-miR-328-3p (human orthologue of rno-miR-328b-3p) is also up-regulated following traumatic brain injury^93^ and its expression in serum was identified as a short-term prognostic indicator following stroke^94^.

Finally, we analysed the inferred mRNA targets of the short-listed miRNAs and identified a set of epileptogenic miRNAs and mRNA targets. Comparison of this filtered network with several publicly available resources, including two relevant human datasets from epilepsy-resected hippocampi, further enriched the network and identified both well-established and novel miRNAs, mRNAs and miRNA-mRNA interactions of translational relevance in epilepsy (Fig. 6).

Our study used a single toxin-free model of epileptogenesis which is highly reproducible and captures many phenotypic aspects of epilepsy. Nevertheless, future studies incorporating different models with different aetiologies will be key to reveal gene pathway changes which are not specific to a given model and/or species. Our dataset contained only male animals. Whilst there is some suggestion that there are no clear sex-specific differences in miRNA dysregulation in human epilepsy, detailed analysis of both male and female animals using our approach may yield sex-specific molecular mechanisms and therapeutic targets at different stages of epileptogenesis. Finally, future wet lab studies could be designed to validate potential therapeutic targets and biomarkers generated by our molecular networks.

In summary, we here generated expression-based temporal Bayesian networks of miRNA-mRNA-mRNA interactions that encapsulate, in an unbiased manner, the complexity of transcriptomic (dys)regulation throughout epileptogenesis. Network analysis and enrichment of network inference with targeted biological information identified novel miRNAs and their mRNA targets which may be closely associated with epileptogenesis, ictogenesis on the day of the first spontaneous seizure, and the early phase of chronic epilepsy, providing new insights into the development of epilepsy and enabling prioritisation for pre-clinical and early clinical therapeutic screening and intervention.

### Supplementary Information

Supplementary Figure 1.

Supplementary Information 1.

Supplementary Information 2.

Supplementary Information 3.

Supplementary Information 4.

Supplementary Information 5.

Supplementary Information 6.

## Data Availability

The datasets used in the study are available in the Gene Expression Omnibus (GEO) under accession number GSE137473. The complete codes for model construction and network analyses are available at www.github.com/nirajkhe/EpimiRNA.
