# Supplementary material for: Integrative network analysis of miRNA-mRNA expression profiles during epileptogenesis in rats reveals therapeutic targets after emergence of first spontaneous seizure
Source: Sci Rep. 2024 Jul 3;14:15313. doi: 10.1038/s41598-024-66117-7 (PMC11222454; doi:10.1038/s41598-024-66117-7)
Supplement: Supplementary file 1 — Supplementary Figure 1. [file 41598_2024_66117_MOESM1_ESM.docx]

**Supplementary Information**

# Integrative network analysis of miRNA-mRNA expression profiles during epileptogenesis in rats reveals therapeutic targets after emergence of first spontaneous seizure

Niraj Khemka^1^, Gareth Morris^2,3^, Laleh Kazemzadeh^1^, Lara S. Costard^4,5^, Valentin Neubert^4,5^, Sebastian Bauer^4,5^, Felix Rosenow^4,5^, Morten T. Venø^6,7^, Jørgen Kjems^6^, David C. Henshall^1,2^, Jochen H.M. Prehn^1,2*^, Niamh M.C. Connolly^1,2*^

^1^Centre for Systems Medicine & Dept. of Physiology & Medical Physics, RCSI University of Medicine and Health Sciences, Dublin, Ireland.

^2^FutureNeuro SFI Research Centre, RCSI University of Medicine and Health Sciences, Dublin, Ireland.

^3^Neuroscience, Physiology and Pharmacology, University College London, London, United Kingdom

^4^Epilepsy Center, Department of Neurology, Philipps University Marburg, Marburg, Germany.

^5^Epilepsy Center Frankfurt Rhine-Main, Neurocenter, University Hospital Frankfurt and Center for Personalized Translational Epilepsy Research, Goethe-University, Frankfurt, Germany.

^6^Interdisciplinary Nanoscience Center, Dept. of Molecular Biology and Genetics, Aarhus University, Aarhus, Denmark.

^7^Omiics ApS, Aarhus, Denmark


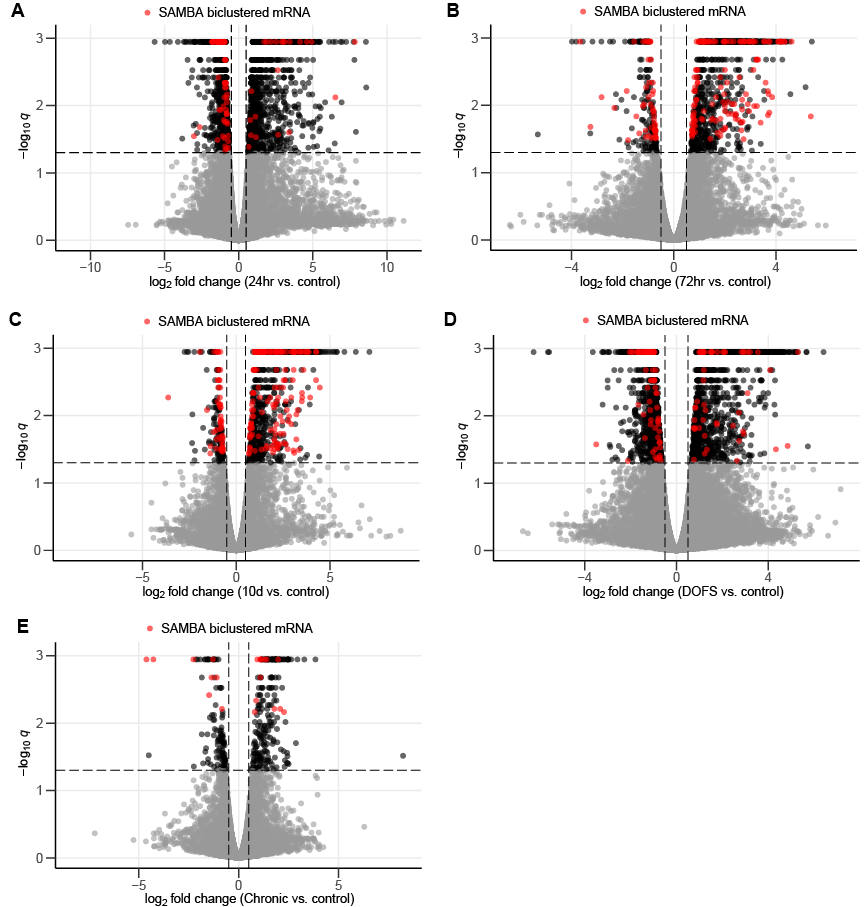


**Supplemental Figure 1: mRNA differential expression analysis (relative to control) across all timepoints.** (A-E) Volcano plots showing log2foldchange (x-axis) against the -log10 adjusted p-value (q; y-axis) for all mRNA relative to control at (A) 24hr, (B) 72hr and (C) 10 day after PPS stimulation, (D) day of first seizure (DOFS) and (E) 1 month after seizure (Chronic). mRNA retained for network generation following SAMBA bi-clustering are coloured red.
